# Effect of Repressing Lithium Disilicate Glass Ceramics on The Shear Bond Strength of Resin Cements

**DOI:** 10.3390/ma16186148

**Published:** 2023-09-10

**Authors:** Tariq S. Abu Haimed, Saeed J. Alzahrani, Esraa A. Attar, Lulwa E. AL-Turki

**Affiliations:** 1 Department of Restorative Dentistry, Faculty of Dentistry, King Abdulaziz University, Jeddah 21589, Saudi Arabia; tabuhaimed@kau.edu.sa (T.S.A.H.); sjalzahrani1@kau.edu.sa (S.J.A.); 2 Department of Oral and Maxillofacial Prosthodontics, Faculty of Dentistry, King Abdulaziz University, Jeddah 22254, Saudi Arabia

**Keywords:** lithium disilicate, heat-pressing, glass-ceramics, repressing, resin cement, shear bond strength

## Abstract

The aim of this study was to investigate the effect of repeated pressing of lithium disilicate ceramic on the shear bond strength (SBS) of three types of resin cement. Methodology: A lithium disilicate ceramic (IPS e.max^®^ Press) was first heat-pressed to form rectangular disk specimens. Then, leftovers were used for the second and third presses. A total of 90 specimens were prepared and separated, according to the number of pressing cycles, into three groups: 1st, 2nd, and 3rd presses (*n* = 30). Each group was further subdivided into three groups (*n* = 10) according to the type of resin cement used, as follows: Multilink N (MN), Variolink Esthetic DC (VDC), and Variolink Esthetic LC (VLC). All the cement was bonded to the ceramic surface, which was etched with hydrofluoric acid and primed with Monobond Plus. All samples were light-cured and stored for 24 h. Shear bond strength was tested on a universal testing machine. Results: A two-way ANOVA was used to evaluate the influence of repeated pressing cycles and cement type as well as their interaction. The results indicated that cement type has a significant impact (*p* < 0.001) but not the number of pressing cycles (*p* = 0.970) or their interaction (*p* = 0.836). The Bonferroni post-hoc test showed that the SBS of MN was significantly higher than that of VDC and VLC in the first press and second press cycles, respectively. The SBS of MN was significantly higher than that of VDC and VLC cements in the third pressing cycle. There was no significant difference in the SBS between VLC and VDC in all three pressing cycles. Conclusion: The results of the current study did not report a detrimental effect of repeated pressing up to three cycles on the shear bond strength of the IPS e.max^®^ Press. Multilink resin cement showed the highest SBS to IPS e.max^®^ Press at the third pressing cycle. For all types of cement and heat pressing cycles, the majority of cement failures were adhesive. No cohesive failures occurred in any of the tested resin cements, regardless of the cement type or the number of heat pressing cycles tested.

## 1. Introduction

Lithium disilicate glass ceramics are known to offer great aesthetic restorations because of their inherent translucency. Lithium disilicate glass ceramic restorations may be processed by the heat pressing technique. IPS e.max^®^ Press was introduced as an esthetic, translucent heat-pressed lithium disilicate ceramic with a 400 MPa flexural strength and a 70% volume fraction of needle-like lithium disilicate crystals [1,2].

Heat-pressed ceramics utilize the lost wax technique with ceramic ingots, which are pressure-pressed into a mold by a plunger in a pneumatic press furnace [3]. Once the lithium disilicate-pressed restoration is divested, the sprue and button portions are discarded, creating a considerable amount of excess wasted/leftover ceramics. Recycling what is usually discarded ceramic is a cost-effective way to reduce the cost of the restoration. The use of repressed lithium disilicate ceramics has been adopted by many investigators [3,4,5,6,7]. Emphases were placed on testing the optical and mechanical properties of the repressed material [6,7,8,9,10]. Albakry et al. [6] and Gorman et al. [7] reported no significant effect of repressing on the biaxial strength of lithium disilicate dental glass ceramics. AbuHaimed et al. [5] reported no significant effect of repeated heat pressing on the flexural strength of lithium disilicate ceramics. In contrast, Chung et al. [8] reported a significant increase in flexural strength as a result of repeated pressing of lithium disilicate glass.

In addition to esthetics, being glass-based and thereby etchable, lithium disilicate ceramics offer the advantages of adhesive cementation by means of resin-based cements. When hydrofluoric acid is applied to dental glassy ceramics, tetrafluorosilane is produced, which further interacts with the glassy ceramic to produce soluble hydrofluorosilicic acid. When the latter is washed away, a high-energy dental ceramic surface with an excellent porous micro-retentive surface is obtained [11,12,13,14]. Adhesion of the ceramic crowns using resin cement was shown to enhance the fracture loads of the ceramic crowns [15]. Da Rosa et al. [16] confirmed the critical role of resin cement chemical adhesion to lithium disilicate in the fatigue behavior of the crown. The authors reported an amplification in stress magnitudes in the crown and cement as a consequence of the gradual failure of the bond [16].

Resin cement is made of an organic matrix with inorganic fillers and an activation initiator. Components of the organic matrix usually include more than one of the following monomers: bisphenol A-glycidyl dimethacrylate (Bis-GMA), triethylene glycol dimethacrylate (TEGDMA), and urethane dimethacrylate (UDMA). Examples of inorganic fillers include barium fluoroaluminoborosilicate, quartz, amorphous silica, and ytterbium fluoride [17,18]. Resin cements are classified as self-cured, light-activated, or dual-activated materials based on the chemical nature of the polymerization initiator [19]. The viscosity of the organic monomer and the amount of initiator contribute to the degree of polymerization of the resin cement. Bisphenol A-glycidyl methacrylate (Bis-GMA) is a large, rigid, and highly viscous monomer [20]. In contrast, the lesser viscosity of the urethane dimethacrylate (UDMA) monomer facilitates the movement of free radicals, thereby enhancing the degree of crosslinking and consequently increasing bond strength [20,21]. Hooshmand et al. [22] reported the important effect of the type of luting cement on the fracture toughness of the lithium disilicate glass ceramic. Marocho et al. [23] reported a significant influence of the type of cement on the microtensile bond strength at the ceramic—resin cement interface.

The bond strength of dental glass ceramics may be affected by differences in the microstructure of the ceramics, the amount of glassy phase, and the size and distribution of crystal sizes after hydrofluoric acid etching [24]. Studies on repressed dental lithium disilicate glass ceramics were in agreement in reporting an increase in the size of the lithium disilicate crystal in the repressed samples [5,6,7,8]. Gorman et al. reported consistent grain growth with increasing the number of heat-pressing cycles for up to four tested heat-pressing cycles. The study reported an enlargement of grain width and length from 0.19 and 1.47 μm to 0.69 and 4.19 μm, respectively, on the fourth heat-pressing cycle. The literature is very deficient in information regarding the bond strength of resin cement to repressed lithium disilicate glass ceramic restoration. It is important to identify the most reliable bonding at the ceramic-resin interface, which relies on the formation of micromechanical interlocking in addition to chemical adhesion.

Information on the shear bond strength of repressed lithium disilicate ceramics to resin cement is deficient, which is crucial for the successful clinical outcome of the restoration. Therefore, the aim of this study was to evaluate the shear bond strength of repressed lithium disilicate IPS e.max^®^ Press to resin cements. We hypothesize that:

1. Repeated pressing of lithium disilicate will not affect the shear bond strength of the repressed samples to the resin cements;
2. The type of resin cement used will not affect the shear bond strength of lithium disilicate glass ceramics, regardless of the number of pressing cycles.

## 2. Materials and Methods

A total of 90 rectangular glass-ceramic specimens were produced and divided into three groups according to the number of heat pressing cycles for the first, second, and third presses, respectively (*n* = 30). Each group was further subdivided into three groups (*n* = 10) according to the type of resin cement used: Multilink N (MN), Variolink Esthetic DC (VDC), and Variolink Esthetic LC (VLC). Table 1 shows the details of the materials used in this study [1,25,26,27,28].

### 2.1. Specimen Preparation

All glass-ceramic specimens were prepared using the lost wax technique. Wax (Renfert, Hilzingen, Germany) was poured into rectangular, custom-made silicon molds with internal dimensions of 8 × 6 mm and a 2 mm thickness. The patterns were then sprued, invested, and heat pressed according to the manufacturer’s recommendations. The process was performed using a pressing furnace (Programat EP3010, Ivoclar Vivadent, Schaan, Liechtenstein). The following furnace settings were used: stand-by and press temperatures of 700 °C and 920 °C, respectively, and a 25 min holding time. A total of ten IPS e.max^®^ Press ingots, shade A1 LT, were used to produce the samples. After pressing, the lithium disilicate glass-ceramic specimens were divested. Later, the sprue and button segments were separated. The leftover sprues and buttons were collected and adjusted as ingots for repressing the second press group. The same process was repeated using the leftover material from the second press to construct samples for the third press group.

**Figure 1:**
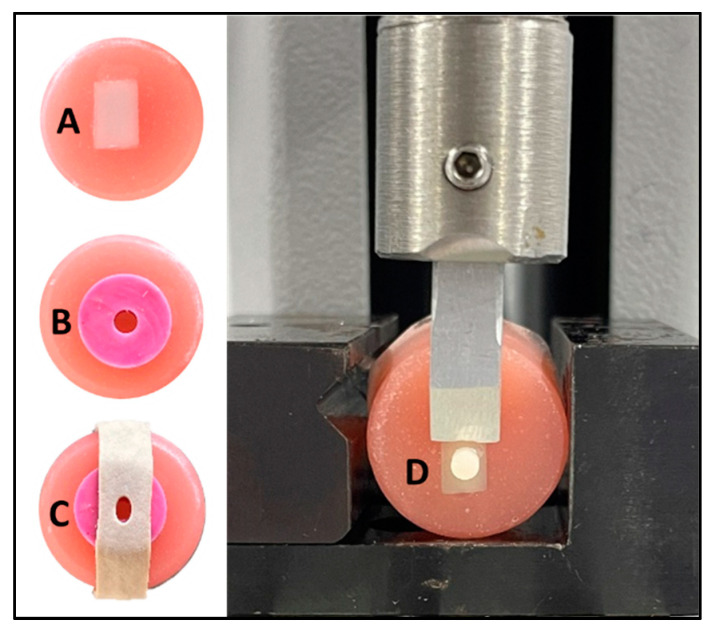
Bonding preparation and testing. (**A**) Shows the ceramic sample embedded in acrylic resin. (**B**,**C**) show the silicone mold before and after fixation with an elastic band. (**D**) Shows the sample during shear testing.

All samples were embedded in acrylic resin molds (ProBase Cold Acrylic Resin, Ivoclar Vivadent, Schaan, Liechtenstein) (Figure 1A), then went through a process of polishing with silicon carbide sandpapers up to 2000 grit in a water-cooled polishing machine (PlanarMet 300, Buehler, Lake Bluff, IL, USA).

### 2.2. Bonding Technique

The same bonding technique was carried out for all resin cements according to the manufacturer’s recommendations. The ceramic surface was etched with 9% hydrofluoric acid for 20 s, washed thoroughly, and dried with an air stream. Monobond Plus primer was then applied for 60 s and dried with an air stream. A specially made circular silicon mold with a 3 mm internal diameter and 3 mm height was secured on the surface of the ceramic with an elastic band (Figure 1B,C). The mold was then filled with each resin cement and light-cured from the top surface for 60 s using Elipar™ DeepCure-S LED Curing Light (3M Unitek, Monrovia, CA, USA). The silicon mold was then gently removed, and an additional 60 s of curing time was performed (top, bottom, right, and left sides). All samples were stored for 24 h until testing.

### 2.3. Shear Bond Strength Testing

A universal testing machine, Multitest 2.5i (Mecmesin, Horsham, UK), was used for SBS evaluation. Each sample was fixed on the lower stage in a vice. A shearing blade was fixed to a 500 N-load cell and moved at 1 mm/min speed until failure (Figure 1D). The SBS was calculated by dividing the load at failure over the surface area of the resin button. 

### 2.4. Interface Failure Analysis and Scanning Electron Microscope imaging

The type of failure was also identified as adhesive, cohesive, or mixed failure under a stereo microscope (RaySmart Technology Co., Ltd., Shenzhen, China) at 50× magnification. A random sample from each pressing group was etched with 9% hydrofluoric acid for 60 s and viewed under the scanning electron microscope Aura100 (Seron Technologies, Gyeonggi-do, Korea) under 5000× magnification.

### 2.5. Statistical Analysis

The SBS values were analyzed for normality and confirmed with Levene’s test (*p* > 0.05). Descriptive statistics of the mean SBS for each press group and cement type were calculated. A two-way analysis of variance (ANOVA) was performed to evaluate the effect of the independent factors (pressing cycles and type of cement) as well as their interaction on the dependent factor (SBS), followed by a Bonferroni post-hoc test [29]. The type of failure analysis was evaluated using the chi-square test [30]. All tests were carried out at a significance level of 5%.

## 3. Results

A summary of the mean SBS distributed by the number of pressing cycles and type of cement is shown in Table 2. The two-way ANOVA indicated that the SBS was significantly associated with the type of cement (*p* < 0.001) but not the number of pressing cycles (*p* = 0.970) nor the interaction between them (*p* = 0.836), i.e., for each cement type, the number of pressing cycles had no impact on the bond strength. However, for each pressing cycle, there was a significant difference in the SBS between cement types.

The Bonferroni post-hoc test results are shown in Table 2. The Bonferroni post-hoc test showed that the SBS of MN was significantly higher than that of VDC in the first press cycles (*p* < 0.05) and VLC in the second press cycle (*p* < 0.05), but higher than both cements in the third pressing cycle (*p* < 0.05). However, there was no significant difference in the SBS between VLC and VDC in all pressing cycles (*p* > 0.05).

**Figure 2:**
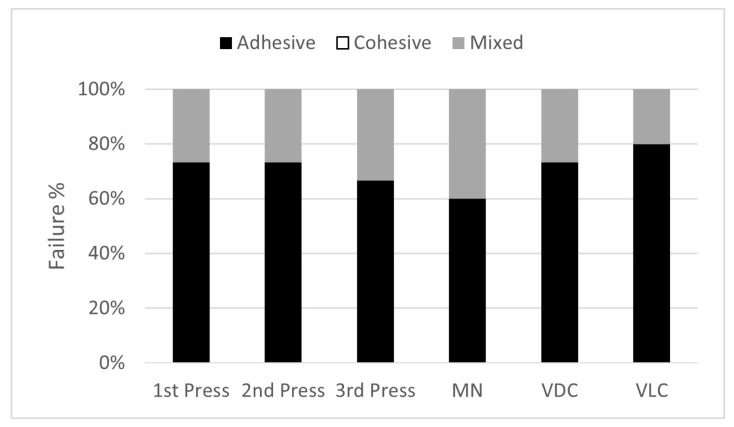
Percentage of each type of failure versus number of pressing cycles and type of cement. There is no significant difference in the type of failure between the number of pressing cycles or cement groups.

**Figure 3:**
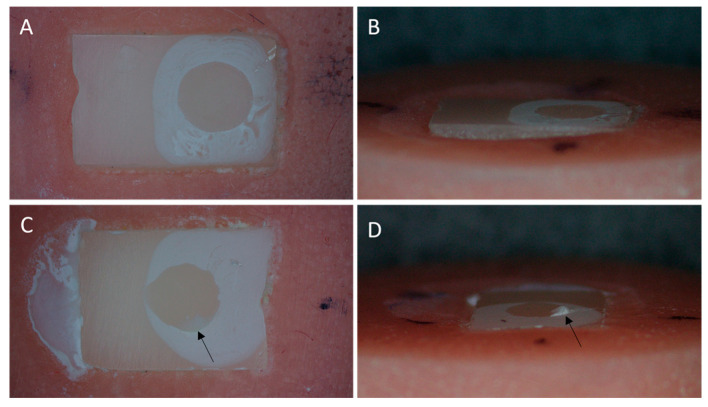
Stereomicroscopic images of the interface of selected failed samples (**A**,**B**) represent adhesive failure. The surface shows no remnant of the cement. (**C**,**D**) represent a mixed failure. A small remnant of cement is apparent at the site of loading (arrows).

Failure interfaces of the failed samples were grouped according to the number of pressing cycles and type of cement, and the failure percentage of each group is shown in Figure 2. Most of the failures were adhesives among all groups. No cohesive failures were reported for any pressing cycle or cement type. A higher number of mixed failure types was associated with the cement MN, regardless of the number of heat pressing cycles. The chi-square test showed no statistical difference between the type of failure and the cement types (*p* = 0.22) or the number of pressing cycles of ceramic (*p* = 0.81). Figure 3 represents an illustration of samples that failed due to adhesive and mixed failures.

**Figure 4:**
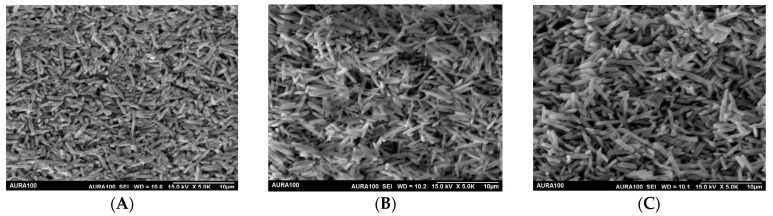
SEM images of pressed IPS e.max^®^ Press samples. (**A**) first press, (**B**) second press, and (**C**) third press. All images were scanned at 5000×. The size of the crystals appears to be smaller and more compact in the first press cycle compared to the second and third press cycles.

SEM images of pressed ceramic samples are presented in Figure 4. All images were taken at 5000× magnification. It is noticeable that the lithium disilicate crystals are less compacted in the 2nd and 3rd press samples, which is associated with growth in the width and size of the crystals.

## 4. Discussion

The current study investigated the effect of the repeated pressing of IPS e.max^®^ Press on shear bond strength in three types of resin cements. The resin cements used were Multilink N (MN), a self-cured resin cement with the option of light curing, Variolink Esthetic DC (VDC), a dual-cured resin cement, and Variolink Esthetic LC (VLC), a light-cured resin cement. The results of this study showed no significant differences in SBS between resin cement and lithium disilicate ceramic regardless of the number of pressing cycles for all resin cements used (*p* = 0.970). Thereby, the first null hypothesis was accepted. The results also showed a significant difference in SBS as a function of the type of resin cement used (*p* < 0.001). Thereby, the second null hypothesis was rejected.

Resin cements are the preferred luting cements for cementation of lithium disilicate ceramics due to the combination of micromechanical and chemical adhesion by means of silane coupling agents [31]. The bond strength of resin cements to dental ceramics may be evaluated by means of shear or microtensile testing. The fabrication of microtensile bond strength specimens is very technique-sensitive and may result in failure of many specimens during fabrication; thereby, shear bond strength was used in this study to evaluate the shear bond strength of repressed lithium disilicate IPS e.max^®^ Press to resin cements. Consequently, ensuring reliable mechanical performance of the ceramic crown.

The bond strength of a resin cement to the ceramic substrate is mediated by several elements relating to the ceramic substrate and the resin cement utilized. Upon etching glassy ceramics, the glassy matrix is selectively eliminated, forming microporosities on the surface of the etched ceramic and exposing the crystalline structures. The exposed crystals act as surfaces that retain resin-cement interlocking, which improves bonding capability [21]. Resin cement flows into surface microporosities to form a strong mechanical bond with the ceramic [19]. The formation of microporosities is affected by the size, quantity, and distribution of ceramic crystals [19,24]. Research on repressed lithium disilicate glass ceramics was in agreement regarding the enlargement of lithium disilicate crystal dimensions in specimens subjected to repressing [5,6,7,8]. AbuHaimed et al. [5] reported an average size of crystals that is about 0.3–0.5 μm and 1–3 μm in diameter and length, respectively, after the first press. In contrast to 0.4–0.8 μm and 2–6 μm in diameter and length, respectively, for the second and third presses. In addition, the authors reported less densely packed crystals of the repressed lithium disilicate. Albakry et al. [6] reported lithium disilicate crystal elongation after repressing Empress 2. Tang et al. [3] reported longer and wider rods. However, the results of the current study did not report any changes in shear bond strength between resin cements and lithium disilicate as a result of repressing, up to three pressing cycles, as evaluated for the tested cement.

The components of the resin cement represent important contributors to the bond strength [32]. Light-cured resin cement depends on photo initiators only for curing in order to provide the operator with the advantage of prolonging working time, a feature that is particularly valuable when handling these technique-sensitive materials. In an attempt to deliver additional polymerization in situations where curing light attenuation may occur, a combination of a chemical, usually an amine, and a photo initiator is used in dual-curing resin cements in varying amounts depending on the cement used [19]. Peutzfeldt [33] reported higher degree of conversion and mechanical properties when dual-cure cements were light-cured compared to purely chemically activated dual-cure cements. Passia et al. [34] emphasized the significant effect of the bonding system utilized on the bond strength of lithium disilicate ceramics. Lise et al. [21] indicated the important contribution of etching the ceramic surface followed by silanization on the bond strength, regardless of the type of resin cement used. Novais et al. [35] evaluated the polymerization and micro-shear bond strength of two dual-cured resin cements (Variolink II and Relay X ARC) using dual activation mode (base and catalyst) and light activation mode (base paste only). In addition to a light-cured resin cement (Variolink Veneer). In the latter study, a significantly higher degree of conversion was reported for the dual cure cement in dual cure mode compared to light curing only [35]. Light-cured resin cement exhibited the least significant degree of conversion [35]. Dual-cure Relay X ARC, used in light activation mode only, exhibited significantly lower shear bond strength than when used in dual-cure mode and even lower but not significantly different from the light-cured Variolink Veneer values [35]. Novais et al.’s [35] study reinforces Passia et al.’s [34] results, signifying the importance of the type of resin cement used. Windle et al. [36] reported the critical role of photoinitiation for both light and dual-cured resin composites and that the self-activation action for the dual-cured composites is dependent on the quantity of light exposure. However, the authors also reported that the depth of cure differed between composites [36]. In the current study, Multilink N exhibited the highest SBS, which was significant after the third cycle. Variolink Esthetic dual cure and Variolink Esthetic light activated showed no significant difference in SBS. The main difference in composition between MN, VDC, and VLC cements is that the size of the filler particles in MN is larger (0.25–0.3 μm) than those of VDC and VLC (0.024–0.02 μm) which may have contributed to the higher strength of the MN bond. The fact that VDC and VLC cements showed comparable values of SBS may be attributed to the design of the study, which relied on a single interface. The light-cured resin cement had direct exposure to the curing light and, therefore, might have resulted in a higher degree of polymerization than if it had been covered by a restoration. Researchers in this study agree with Lopes et al. [28] in that since the exact composition and concentrations of resin cements are not clearly identifiable, explanations of research results related to resin cement components are speculative. 

To our knowledge, only one study evaluated the effect of repressing on the shear bond strength of lithium disilicate ceramics. The results of the current study do not agree with El-Etreby et al.’s [37] results. In El-Etreby et al.’s [37] study, repressed and etched lithium disilicate showed statistically higher shear bond strength values than pressed and etched.

When a ceramic restoration is bonded to the tooth structure, two distinct interfaces are created. Ceramic–resin–cement interface and resin cement–tooth interface [19]. The bond strength of a resin cement at the cement–ceramic interface may not be the same as the bond strength at the cement–tooth structure interface. A strong ceramic–cement interface bond may be affected by tooth–resin–cement interface bond strength, and vice versa. Aker Sagen et al. [38] evaluated the shear bond strength of lithium disilicate ceramic to dentin. In the latter study, no significant difference in bond strength was noticed between Multilink and Variolink esthetic resin cements. Most Multilink failures were cohesive within the cement, followed by combination failures and adhesive–cement–ceramic failures, respectively. There were no adhesive failures at the Multilink resin–cement–dentin interface. In contrast, Variolink esthetic failures were mostly adhesive failures at the ceramic-cement interface, followed by adhesive failures at the ceramic–dentin interface. On the contrary, Marocho et al. [23] reported stronger bonds at the resin–cement–ceramic interface than at the resin cement–dentin interface and cautioned regarding the interpretation of the evaluation of resin cement’s performance. In the current study, only the ceramic–resin cement interface was tested to exclude any possible contribution of the resin cement–tooth structure interface. The results of the current study did not demonstrate cohesive failures within the cement, regardless of the number of pressing cycles or the type of cement. Most failures were adhesive at the resin–cement–ceramic interface, followed by mixed failures for all pressing cycles and for all resin cement types. The majority of mixed failures showed a crescent of cement at the site of shear loading. However, this may be attributed to the nature of shear loading, which was found to have an initial tensile stress [39]. Therefore, the force applied to the substrate could cohesively disrupt the substrate before shearing the bond, leading to a small piece of cement left on the substrate. Nevertheless, the SBS was uniformly distributed across the samples that were labeled as adhesive or mixed failures. 

## 5. Conclusions

Repressing IPS e.max^®^ Press did not display a significant effect on shear bonding to resin cements, regardless of the number of pressing cycles. The type of resin cement used exhibited a significant influence on shear bonding to the IPS e.max^®^ Press. Multilink resin cement used in dual cure mode showed significantly higher shear bond strength values to IPS e.max^®^ Press on the third pressing cycle. Most cement failures were adhesive for all cement types and heat pressing cycles. No cohesive failures occurred in any of the tested resin cements, regardless of the cement type or the number of heat pressing cycles tested.

## Figures and Tables

**Table 1:** Materials used in the study.

Material	Description	Manufacturer
IPS e.max^®^ Press	Lithium disilicate (Li_2_Si_2_O_5_): ≅70% volume of needle-like lithium disilicate crystals in a glassy matrix.	Ivoclar Vivadent, Schaan, Liechtenstein
Multilink N. Dual cure	Monomer: Bis-EMA, UDMA, Bis-GMA, and HEMA. Inorganic fillers: ≅40% by volume of barium glass, ytterbium trifluoride and spheroid mixed oxide. Particle size is 0.25–3.0 µm, with a mean particle size of 0.9 µm.	Ivoclar Vivadent, Schaan, Liechtenstein
Variolink Esthic DC. Dual cure	Monomer: UDMA and methacrylate monomers. Inorganic fillers: ≅38% by volume of ytterbium trifluoride and spheroid mixed oxide. Particle size is 0.04–0.2 μm, with a mean particle size of 0.1 µm.	Ivoclar Vivadent, Schaan, Liechtenstein
Variolink Esthic LC. Light cure	Monomer: UDMA and methacrylate monomers. Inorganic fillers: ≅38% by volume of ytterbium trifluoride and spheroid mixed oxide. Particle size is 0.04–0.2 μm	Ivoclar Vivadent, Schaan, Liechtenstein
Monobond Plus Primer	Silane methacrylate, phosphoric methacrylate, and sulfide methacrylate	Ivoclar Vivadent, Schaan, Liechtenstein
Porcelain Etch	9% hydrofluoric acid	Ultradent, South Jordan, UT, USA

**Table 2:** Mean and standard deviation of SBS and Bonferroni post-hoc test results. Superscript letters indicate significant differences among columns (cement type).

		SBS MPa (SD)	
Cement Type	1st Press	2nd Press	3rd Press
MN	18.08 (1.96) ^A^	18.15 (3.54) ^A^	18.48 (3.49) ^A^
VDC	14.02 (3.84) ^B^	15.18 (3.41) ^A^	14.68 (2.34) ^B^
VLC	15.01 (3.81) ^A^	13.70 (3.58) ^B^	14.45 (3.69) ^B^
Post-hoc pairs with Bonferroni adjustment	MN-VDC = 0.012 MN-VLC = 0.082 VDC-VLC = 1.0	MN-VDC = 0.128 MN-VLC = 0.008 VDC-VLC = 0.960	MN-VDC = 0.036 MN-VLC = 0.023 VDC-VLC = 1.0

Overall *p* value for the number of pressing cycles (*p* = 0.970) and cement type (*p* < 0.001).

## Data Availability

The data is available on request.
